# Practical Illustrations of Typhus, and Other Febrile Diseases

**Published:** 1817-01

**Authors:** 


*Practical Illustrations of Typhus, and other Febrile Diseases.* By JOHN ARMSTRONG, M. D. Octavo, pp. 306. London, 1816

IF an impulse be given to a quiescent pendulum, its eccentricities, however great at first, will be gradually and constantly curbed by the power of gravitation, till its vibrations cease, and it hangs in *equilibrio,* obedient to the laws which govern surrounding matter and the earth itself. So, TIME, in medicine, like gravity in the above instance, is progressively, though slowly, checking the aberrations of theory, and drawing the opinions and practices of the votaries of the Healing Art into greater unison with Nature, with truth, and with each other. True it is, that in every generation, the effulgence of some genius, or the influence of some fashion, gives a powerful shock to the medical pendulum, and numerous vibrations are necessary before the balance of sober reason is restored. Yet, amidst these vicissitudes, the march of medical science, though silent, is progressive; the materials of a lasting edifice are accumulating; the arch of triumph is rising; and the Phœnix of Truth is daily expanding her golden wings over the ashes of error. In medicine, as in other sciences, from collisions of opinion, intellectual scintillations are constantly emanating that throw gleams of light on phænomena previously involved in Cimmerian darkness; and such is the wisdom of Providence, that the most preposterous doctrine, the most pernicious practice, carries with it not only a salutary principle of self-correction, but a useful beacon to warn others of their danger. If, in the wrecks of theory, the fatalities of experiment, and the failures of speculation, thousands have perished, these very mementos of our weakness form so many imperishable land-marks to guide us through those syrtes, rocks, and whirlpools so numerously scattered throughout the ocean of Medical Literature.

These observations are peculiarly applicable to the subject of the volume before us. A disease which, in its extended signification, has been calculated to occasion seven-eighths of our mortality; which is common to both sexes; to all periods of life; and to every climate and country, must have excited the attention of poets, philosophers, and physicians from the remotest antiquity. Yet strange as it may seem, from its *first recorded* ravages in the Grecian camp on the banks of the Scammander, down to its direful diffusion among our fever-stricken legions on the shores of the Scheldt; after all the melancholy experience that has been gained in every parallel of longitude between the Missisippi and the Ganges, from Iceland to the Cape, the nature and treatment of this wonderful disease should still be involved in such obscurity as to yearly, and almost monthly, excite discussion, and give birth to volumes on the subject! Hence it might appear to superficial observation, that on this point we had made little progress; but this is not the case. The circle of litigation is greatly narrowed; theories are now drawn from facts instead of imagination. Although the modes of treatment differ, it is more in degree than in principle; and, in fine, the causes of fever are now better understood; its *ratio symptomatum* more clearly developed, and the practice more successful, than in the time of our forefathers, or even in our own early days.

The great improvement of late years in the treatment of fever appears to spring from more accurately discriminating *cause from effect*, and in tracing, by means of morbid anatomy, those lesions which are connected with the progress of fever, whether as one or the other. The terrible and destructive warfare too, in which we have been engaged during the last twenty years, has given opportunities innumerable for men of genius and talents to burst the trammels of scholastic dreams and errors; and to dissipate those illusions and preconceptions which held the heads, hearts, and hands of medical men spell-bound in prejudice, timidity, and inaction. If in these cases, *extremes* have occasionally been run into, what has temerity done, compared with what timidity has undone? If in the last few years evacuations have sometimes been carried to excess; what mischief has resulted, compared with the effects of excessive stimulation, in the preceding years? Have the lancet, calomel, and jalap as many murders to answer for as bark, wine, and opium? We believe not. Yet the ancient edifice of error and prejudice is by no means demolished. We every day, and almost every hour, see victims immolated at its altars. The doctrines of *debility* having spread from the profession to the community at large, they here took fast root, since they corresponded exactly with superficial observation and self-indulgence. Sickness of every kind is necessarily accompanied by a sense of *weakness*; and as the vulgar have no idea of acquiring *strength* in any other way than through food, drink, or medicine, the two first are diligently applied, and the last must be of a cordial and tonic nature of course. The proposition of curing *debility* by bleeding, purging, and low diet is very far beyond their comprehension; and we are sorry to say beyond the comprehension of many who ought to know better. It was, therefore, with more than usual anticipations of pleasure, that we opened the work of Dr. Armstrong, knowing as we did, that "nullum tetigit quod non ornavit."

The work of Dr. Parry enriched our first Volume, and there shone—"velut inter ignes, Luna minores." The work before us is scarcely inferior in point of value, though less comprehensive in object. It stamps the reputation of its author with a character so indelible, that

Time, which soon washes away all subordinate impressions, will only render its features more conspicuous. Like the writings of that excellent pathologist above-mentioned, Dr. Armstrong's book defies condensation, so concentrated is the knowledge, and so valuable is the matter in every page. This, however, is of little consequence, since we are pretty confident, that from the specimens which we shall adduce, no man who studies his profession with zeal, will hesitate or delay making himself acquainted with every line of the original.

Dr. A. divides the pyrexiæ into three orders, as arising from contagion, miasmata, and topical affections.

"In the two former, the fever cannot be traced," says he, "in the first instance, to any single derangement, but rather seems the product of many morbid actions existing at the same time."

In this he differs from the Clutterbuck theory; and his sentiments are congenial with our own, as is evident in various parts of this Journal.

*Typhus.* Our author justly observes, that Typhus is often "made to include a great variety of widely different affections; especially, when in their course, the concomitant fever puts on a low or putrid type, as frequently happens in the last stages of many inflammatory diseases of the external and internal textures." Dr. A. therefore, restricts the term to the peculiar disease so well known in this country, as arising from specific contagion, and possessing the power of propagation.

"The word Typhus, is still too generally associated with the opinion, that the fever, which it properly designates, is in all its stages, a disease of real debility; but this notion has either been taken on the word of those authorities, who for a time gave the tone to medical opinions and theories, or it has been impressed upon the minds of those who retain it, from a contemplation of the disorder wholly limited to its advanced periods. An extensive observation, however, during a series of years, has convinced me, that the genuine typhus, so far from being of an asthenic nature, is most certainly an affection of excitement or of congestion, in its first stages, demanding at such times the decidedly evacuant plan. Entertaining these sentiments, the professional reader will, perhaps, excuse their brief annunciation in entering on the subject; as they may contribute to warn him against the undue influence of some early associations, and thus incline him to give a fair and full hearing to the facts and arguments about to be adduced."

Dr. Armstrong thinks, that there is just ground for dividing typhus into three varieties; namely, the simple, the inflammatory, and the congestive. These he describes and characterizes with a minuteness, a fidelity, a precision, that shews with what accuracy, patience, and discrimination he must have observed and treasured up in his mind, those fleeting phænomena of disease which too often are passed unnoticed by the routine physician. We can only give a rapid sketch of the principal features in these descriptions.

1. *Simple Typhus*, has a first stage of oppression, a second of excitement, and a third of collapse, bearing a ratio as to degree, but not as to duration. The stage of oppression displays paleness of the face; dejection; darkness or livor round the eyes; prostration of strength; diminished mental energy and insensibility; alternate heats and chills; nausea or vomiting; weight and anxiety at the præcordia; sighing, and hurried breathing; heaviness or giddiness of the head; coldness of back; pains in the loins; quick, low, struggling pulse, irregular as to frequency and force. The developement of this stage is sometimes rapid, generally insidious, occupying a period of two or three days, when the second, or stage of excitement, is ushered in.

"The tone and velocity of the circulation is now preternaturally increased; and the pulse accordingly becomes comparatively expansive, thrilly, and resisting; at least, it is widely different from the variable, confined, inelastic pulse of the former stage, and from the uniform, free, and smoothly flowing one of health. The cheeks are flushed; the eyes heavy; and the lips parched. The respiration is quick; the skin almost invariably dry; the heat universally diffused, and steadily above the common point; the tongue foul; the thirst urgent; the uneasiness in the head increased; the sensorium in a highly susceptible state: every symptom, in fine, denoting an exce-s of excitement. Thi second stage of the simple typhus naturally holds a tolerably even tenor for some time. As it proceeds, however, the brain, at intervals, is usually disturbed with reverie or slight delirium, coming on towards the evening, when there is an exacerbation of the fever, and receding towards morning, when there is a remission; but the prostration of strength, which is at all times very evident, is generally greatest in the periods of the exacerbations. During the predominance of the excitement, the bowels, for the most part, have a tendency to constipation. The excretions as well as secretions, also undergo gradual and material changes which are evinced by the dark and offensive nature of the fæces, the peculiar odour of the breath and whole body; and by the morbid appearances exhibited on the tongue, in the fluids formed from the liver, kidnies, and other organs of secretion." p. 11-12.

After six or seven days, sometimes more, sometimes less, excitement glides into collapse; announced by depression of the voluntary powers; relaxation of the surface; variable and lower temperature; diminished force in the circulation; pulse smaller, softer, more undulating. In mild cases, this change indicates convalescence. The tongue will now be found softer and cleaner; the thirst diminished; the breathing deeper; the skin more natural. The functions of the stomach will be, in some degree, restored; the appearance of the fæces improved; a lateritious sediment in the urine.

But in more dangerous cases of simple typhus,

"The supervention of the stage of collapse considerably augments the danger: for the prostration of strength then becomes far greater; the pulse commonly quicker, and always much weaker; the tongue fouler, darker, and drier; the voice fainter, and the articulation less distinct; the respiration shorter, feebler, and more anxious; the sensorial functions more disordered; and the countenance more dejected, sunk, and inanimate. Added to these symptoms, the skin feels looser, and appears more shrivelled; while the temperature is no where so intense as in the stage of excitement, but variable in the course of the day, even on the central parts: and there is an increase of general restlessness; a more perceptible and peculiar fætor about the body; and often an irritating species of cough, which comes, as it were, in convulsive fits. In this state, the patient is disposed to lie upon his back; and, as the peril increases, not only labours under subsultus tendinum, visual deceptions, low muttering delirium, and difficulty of deglutition, but has also a tendency to slide downwards in the bed, and to draw up the feet frequently towards the body." p. 13.

The above are *unfavourable* specimens of the condensed style and invaluable matter with which every page of this work is pregnant. There is but one modern work which will bear a comparison with the present—it is Dr. Parry's Elements of Pathology. These two will stand eternal monuments of fame to their authors, when the marble tombs that enclose their ashes shall have mouldered into dust; and not a stone shall tell where they lie.

In fatal cases of the foregoing variety of typhus, "dissection commonly reveals slight remains of irregular determinations of the sanguiferous system, in some of the viscera or their investing membranes. But whatever proofs of a previous over-excitement may be discovered, there is hardly ever any *visible* disorganization; at least, my own anatomical investigations would lead me to draw this conclusion." p. 14. Dr. A. thinks, that these slight *post mortem* appearances do not satisfactorily account for the unfavourable issues; "and we are almost compelled to infer, that it principally depended upon an actual exhaustion of the vital principle, induced by the preceding excitation." *ib.* In this we fully agree with our author. He acknowledges, indeed, that in certain fine tissues and structures, such as the brain or its membranes, lesion may be produced during the high-tide of fever, which may escape anatomical cognizance. We recommend the following passage to be printed in letters of gold, and hung up in the library of every physician; in the surgery of every surgeon; and in the shop of every apothecary, to be read over once a week in the fever-season of every year.

"In the first stage of the simple typhus, the debility is merely *apparent*, and chiefly dependent on the preternatural accumulations of blood in the vessels about the head, heart, liver, and other internal parts, while there is less circulating upon the surface of the body, than in a natural state. In the second stage, *the debility is still only apparent*, being then the consequence of over-excitement of the heart and arteries; but in the third and last stage, beyond all dispute, the debility is real, as it is then connected with a general collapse, which sooner or later succeeds to a state of febrile excitement, as certainly as exhaustion follows a fit of intoxication. Those who have adopted the beautiful, but deceptive doctrines of Dr. John Brown, will on no account admit, that the debility of typhus is only seeming, during the greater part of its progress. In truth, the impartial observer must confess, that this proposition is not evident at first sight; but, nevertheless, it is as certainly true as any in physic, *as is proved from the debility, in the first and second stages, being increased by wine and cordials, and lessened by evacuations.*" p. 18.

Although Dr. A. allows, that the first appearances of derangement in fever manifest themselves in the nervous system;

"Yet, if any particular system," says he, "be more affected than another, it is unquestionably the sanguiferous, through which the *permanent* effects of fever are chiefly to be traced, and by which the state of the brain and nerves at least, seem [seems] eventually regulated. Nay, if we consider the degrees of visceral congestion, which accompany the whole stage of oppression, we may perhaps find sufficient data for concluding, that the nervous appearances, even from the very first attack, are only secondary of vascular disorder. So great indeed, in a practical point of view, is the importance of attending to the state of the circulation in febrile complaints, that guarding against determinations of blood to the different viscera, and removing preternatural accumulations, whether congestive or inflammatory, when they actually take place, will be found to constitute one of the grand secrets of successful treatment." p. 20.

Dr. A. acknowledges the difficulty of distinguishing simple excitement of the circulation from actual inflammation; yet as the latter frequently grows out of the former, we should be constantly on our guard; "and day after day, make the most scrupulous inquiries, in order to detect, and if possible to arrest, the very first appearances of inflammation supervening in a vital quarter." p. 21. In a note, Dr. A. throws out a hint, that the relations existing between the arterial and venous systems, in many acute and chronic diseases, have not been sufficiently attended to. One of the Editors of this Journal has the subject under consideration, and will probably throw some light upon it.

"*Inflammatory Typhus.* In pleurisy, and similar disorders, the seat of the fever is always local, its effects general, and its nature inflammatory; but some ingenious authors, with Ploucquet and Clutterbuck at their head, seem to me to have proceeded too far, in asserting the same thing of what are called idiopathic fevers. As an example in point, typhus undoubtedly sometimes begins and terminates without topical inflammation; and as inflammation may occur in one or more points, without ever producing an infectious distemper with the true characteristics of typhus, it is evident, that inflammation is not its inseparable and essential constituent. When, therefore, this peculiar disease and inflammation are combined together, it appears only reasonable to conclude, that the latter may have been produced by cold, or any other common cause of fever, operating with the contagion: or that it may have arisen as an effect of the excitement of the heart and arteries, favoured by some predisposition to inflammation in the part affected; as an inflammatory affection of the chest often arises in the measles, though each may exist independently of the other. Some authors have contended, that the inflammation, which accompanies the complicated forms of typhus, occurs with the fever, or even precedes it; and thus, that it merely follows as a consequence of the general excitement. According to my observations, the local inflammation occasionally commences as soon as the fever itself, but generally arises during the stage of excitement; and hence, perhaps, it may be fairly inferred, that, for the most part, it stands in the relation of an effect, rather than a cause of the fever. If we reflect, that more or less visceral congestion attends the first stage, it will not seem improbable, that, by a preternatural distention of the vessels, it may leave a morbid tendency in some organ or other, which may pass into inflammation by the excitement of the second stage. It usually happens in new symptomatic fevers, that the inflammation is limited to one part in particular; but this does not so generally obtain in typhus; for though, one organ may exhibit by far the strongest evidences of inflammation, some other part will often be affected in a less degree; which surely favours the notion, that the topical disorders are commonly the products of the general excitement. But the mode in which inflammation is produced in typhus, it is not so important to investigate, as at what time it takes place, what characters it assumes, and in what parts it is seated." p. 24.

Our author considers the brain, lungs, stomach, liver, and intestines, as the organs on which, in typhus, both the acute and sub-acute forms of inflammation, principally fall. The acute form begins to develope itself on the first, second, or third day of the *second stage*. The sub-acute after the third day of the second stage; and it is less clearly marked:—evinced rather by uneasiness in particular regions, and derangements in particular functions, than by the violent pain and palpable symptoms of the acute form.

"So far as my remarks have extended, the brain and its investing membranes are more subject to inflammation in typhus, than any other parts of the system. When the acute form of inflammation exists within the head, it is generally marked by great mental and corporeal irritability; an anxious, oppressed, or intoxicated cast of the countenance; dry, foul tongue; quick, vibratory pulse; flushed, turgid face; deep pulsating pain in the head; increased heat of the forehead, temples, and hairy scalp; throbbing of the carotid arteries; tinnitus aurium; redness, rolling, and morbid sensibility of the eyes; and more or less disorder in some of the external senses. There are generally transient pains in the limbs; oppression of the præcordia; torpidity of the intestines; uneasy respiration, attended with heavy sighs; nausea, retching, or vomiting, augmented on motion; fretfulness and jactitation; watchfulness; confusion of mind; visual illusions, and high delirium, follow each other in quick succession. If the inflammation should uninterruptedly advance, to these symptoms succeed, indifference to surrounding objects; faultering, or imperfection of the speech; gradually increasing stupor; bloatedness of the face; brown or black parched tongue; low mutterings; tremors of the hands; stupid, suffused, watery eye; squinting or dilatation of the pupil; paralysis of one of the palpebræ; vibices, or petechiæ; oozings of dark blood from the mouth and nostrils; stertorous breathing; general convulsions; relaxations of the sphincter muscles; and other mortal signs." p. 27.

When the brain is thus actively inflamed, the mortal issue is sometimes very rapid: three or four days from the commencement of excitement; but generally protracted beyond the first week. The following, or sub-acute inflammation, however, is more common than the preceding; and we cannot convey a better idea of it to our readers than in Dr. Armstrong's own energetic language.

"For some days, the sub-acute inflammation of the brain most frequently steals on in typhus, by almost imperceptible approaches. At first there are little more than the usual degrees of head-ache and of vertigo, with general lassitude; fugitive pains in the muscles or joints; torpid bowels; and uneasy feeling at the pit of the stomach, commonly accompanied with loathing of food, and a disposition to vomiting; especially on any sudden change of posture. The pulse is small and quick; but the carotid, and even the temporal arteries, beat with rather more than ordinary force. The tongue at first is covered with a whitish fur; the cheeks are alternately flushed and pale, throughout the day; the countenance has a heavy, wearied expression; and the eyes often feel uneasy, as if small particles of sand were in them. Besides, some of the rest of the external senses are almost always disordered; particularly the hearing; which, though occasionally more obtuse, is generally more acute than natural. The fore-mentioned symptoms continue without material alteration for three or four days; although the patient may often be remarked to sigh, breathe quicker, and grow more irritable as well as restless, seldom remaining long in the same place or position. At length, pain of the head, and uneasiness in the orbits of the eyes are more severely felt; the eyebrows are sometimes suddenly knit together; the arms tossed about the bed; or one or both hands now and then pressed against the forehead. The pain of the head continues to increase; and in two or three days more, there are sensations of an indescribable uneasiness, constantly and distinctly referred to the brain. The eyes are now rather blood-shot, and intolerant of light; the anxiety of the præcordia is much augmented; the respiration more hurried; the heat of the surface more elevated; the face permanently flushed; the tongue drier and stiffer; and the involuntary sighing more frequent. The patient now lies at night with his eye-lids half closed in light indistinct dozings, associated with moaning; frightful dreams, and startings; or he is harassed by perpetual watchfulness, joined with frequent wanderings of the mind. As the inflammatory affection advances, day after day the sensorial functions continue to be more and more disturbed. At last delirium becomes unceasing; when signs of an oppressed brain gradually make their appearance; under which the patient slowly sinks into dissolution, with hiccup, petechiæ, subsultus tendinum, an apoplectic expression of the features, and a red, glary eye, floating insensibly in an envelope of mucus."—p. 30.

This species of typhoid inflammation of the brain may run a course of thirteen or twenty days, exhibiting a considerable variety of expression. Nearly the same morbid appearances of the brain or meninges present themselves *post mortem* in both species. The pia or dura mater almost always shews marks of increased vascular excitement, with effusion of coagulable lymph between them; adhesions between the convolutions and hemispheres; red points in the medullary substance; choroid plexus turgid with blood; serous fluid in the ventricles.

In lax irritable habits, hysterical women, and inebriated constitutions (if we are allowed the expression) the simple typhus is occasionally accompanied with fits of almost maniacal delirium; transient flushes of the face, followed by pallor; weak, variable pulse; light tremors of the hands; softness, and dewy moisture of the skin; little augmentation of heat; hurried, unconnected speech; earnestness in the pursuit of a variety of imagined objects. These, Dr. A. thinks, mark a very different state of the brain from that connected with the foregoing modifications of typhus.

In cold, variable weather, the lungs and their connections suffer, sometimes from the acute, more frequently the subacute inflammation. In the former case there will be, conjoined with the ordinary symptoms of typhus, permanent pain in some part of the chest, acute or obtuse, but always increased by inspiration; weight or constriction across the chest; laborious respiration; motion of the alæ nasi; restlessness; cough; features indicative of surprize, alarm, or anxiety: eyes prominent; cheeks and lips redder than natural; face sometimes pale and bloated; pulse variable, as also the temperature.

Dr. A. goes on to describe the complications of typhus with particular portions of the thoracic and abdominal contents; in all of which descriptions the hand of a master is every where apparent. *Congestive typhus* next occupies our Author's attention. In the more violent forms of these, the stage of excitement rarely shews itself at all, unless brought forward by art; the energies of the system seem overwhelmed, or nearly annihilated, by the visceral congestions, and incapable of general reaction. The common series of febrile phænomena is here broken; there is either total want of heat, or local concentration of it from partial reactions. Like the other forms, but more dangerous, it tends to depress the functions or derange the structure of some important organ, "by an almost stagnant accumulation of blood in some part of the venous system." 68. For the admirable symptomatology of this form of fever, we refer to the work itself. The limited opportunities of opening fever subjects, and especially contagious fevers, have prevented the morbid anatomy of these diseases from being so fully investigated as might be desirable; Dr. A. thinks, however, from what he has seen, that the brain and liver bear the onus of disease in congestive typhus.

"It has been noticed, (says our author) that a distended state of the venous system exists in the first stage of the simple typhus; yet so slight, as to give way to the occurrence of the stage of excitement, which comparatively equalizes the circulation. The congestive differs from the simple typhus: first, because the viscera are far more engorged in the first stage; and secondly, because through the continuance of the engorgement, that stage is followed by a general collapse, without the intermediate one of regular and universal excitement, which not only partly characterises the simple typhus, but produces the occasional and partial congestions of its last stage." 75.

After this follow many admirable reflections and observations on the derangements of balance in the venous and arterial systems. We think we can predict with some certainty, that a new light will shortly be shed on this subject through the medium of this Journal, which will explain many phænomena, and confirm many suspicions brought forward by Dr. Armstrong. At page 78, Dr. A. queries in a note,

"Can this viscus (the spleen) be intended by nature as a receptacle for venous blood on those emergencies which are liable greatly to disorder the circulation; and do its structure and situation seem fitted for such purpose?"

We answer, that we have long looked upon it in that light, but conceive the whole circle or set of vessels composing the vena portarum officiates in the same way upon these occasions.

"The most violent forms of the congestive typhus often resemble apoplexy in their symptoms, to which, indeed, they often have a near affinity in their pathology. The balance between the arterial and venous, systems, is more or less disturbed in every instance of congestive fever; for there is more blood accumulated in the veins, and of course, less contained in the arteries than in a natural state. This loss of balance is especially observable on the skin, less blood circulating in the vessels of that part than common, while the central organs of the body are greatly engorged. It is perhaps, to the preternatural fulness of the larger veins, that the lowness and oppression of the pulse ought to be attributed; at least it generally rises after depletion from the veins, which seems to restore the circulation to an equal state again. From observation and dissection, I am certain, that venous congestion exists in many acute and chronic diseases, combined with a deficiency of arterial action; and that in such cases, contrary to the common opinion, a low, feeble pulse indicates, in the first instance, the propriety of depletion, rather than that of stimulation." p. 79.

Dr. Armstrong next draws a parallel between typhus and the plague, as described by Russel, Faulkner, and others; from whence he is led to conclude, "that a close analogy actually does exist between these two maladies," and, that plague "is not a disease of real debility, but one of excitement and congestion." 82. The *post mortem* appearances, Dr. A. thinks, confirm this opinion; as marks of inflammation or congestion are generally found in one or more of the internal viscera. Our author here takes leave to differ from Dr. Pearson, who, by way of lessening the force of these lesions,

"Affirms, that what he calls *pestilential*, is very different from *simple* inflammation, not being accompanied by a hard pulse, nor by an exudation of coagulable lymph, but rapidly terminating in gangrene."

Dr. A. justly observes, that it does not legitimately follow from these circumstances that the inflammation is essentially different from simple inflammation, concerning which similar assertions may be made; as, for instance, in simple inflammation of the intestines, where neither hard pulse nor exudation of coagulable lymph precede mortification. But leaving the symptoms and dissections, the opinions and practices of the ancients, and some moderns, tend to countenance the idea of the plague being inflammatory or congestive. Bleeding has been employed in the East, from the earliest records. Oribasius scarified his own legs when seized with the plague, and abstracted two pounds of blood, by which he was cured. The like practice, he says, succeeded with others. In the time of Russel, bleeding was still had recourse to by the Asiatic physicians as well as by himself. Sydenham quotes fourteen authors of note who recommend blood-letting, while he himself eulogizes the practice, considering the plague like erysipelas, a most inflammatory affection. Mead is of the same opinion.

"If we impartially investigate the histories of the plague, as it has prevailed at different times, we shall almost uniformly find, that the cordial plan of treatment has been attended with the most disastrous consequences in severe cases; and, contrasting these with the good effects repeatedly produced by opportune and free depletion, it will surely be admitted, that we ought not now to be deterred from giving the latter method the fairest and fullest trial, because half measures have so often failed, or because the modern doctrines of debility have paralyzed the right arm of many European practitioners. Indeed, it will be evident to every attentive observer, that in those violent forms of the plague, which terminate in a very short time, the purely stimulant treatment cannot possibly be of essential service, partly on account of its general inefficacy in the beginning of all acute fevers, and partly on account of the dangerous character of the particular disease; and to neglect so powerful a remedy as depletiou, in such cases, is in effect, to leave the unfortunate patients to struggle unassisted with their fate; like those inhabitants of certain countries in the East, who are said to be deserted by their brethren in the extremities of age and sickness. But it may be presumed, that the example which some practitioners have so successfully set as to the treatment of the yellow fever, will be followed by those who may hereafter witness the plague; and we shall then probably find, that the latter disease, like the former, will cease to be so generally fatal, when the palliative plan is abandoned for decisive measures at the commencement." p. 96.

Dr. Armstrong here throws out some hints respecting epidemic and sporadic fevers. Although he allows that the characters of epidemics assume features considerably different; yet he thinks that it is in the medical as in the moral world, similar effects may be produced by different causes; and he means distinctly to say,

"That beside their peculiarity, epidemics generally have such external phænomena, and generally produce such internal derangements, as to prove them to be attended with a simple, an inflammatory, or a congestive fever, by the character of which the treatment must be mainly regulated, however various their abstract nature or origin."

In these sentiments we fully agree; and often have we regretted and wondered to see men, even of talents and experience, prescribe for a name, instead of endeavouring to obviate the *effects* of fever. These effects will be found under the different appearances described by Dr. Armstrong, whatever may be the name or nature of the fever; whether it be produced by the contagion of Cairo; the palludal effluvium of Batavia; or the infectious miasma of typhus.

We shall conclude in our next number, by unfolding a sketch of Dr. Armstrong's modes of treatment, in the three forms of fever above described. In the mean time we most emphatically urge every lover of truth, and every one who cultivates the healing art with zeal, to place the original where he can

"Read it by day, and study it by night."

